# Remote Performance Modulation of Ultrafiltration Membranes by Magnetically and Thermally Responsive Polymer Chains

**DOI:** 10.3390/membranes11050340

**Published:** 2021-05-04

**Authors:** Arijit Sengupta, Anh Vu, Xianghong Qian, S. Ranil Wickramasinghe

**Affiliations:** 1 Ralph E Martin Department of Chemical Engineering, University of Arkansas, Fayettteville, AR 72701, USA; arijita@barc.gov.in (A.S.); anhtvu1985@gmail.com (A.V.); 2 Bhabha Atomic Research Centre, Radiochemistry Division, Mumbai 400085, India; 3 Department of Biomedical Engineering, University of Arkansas, Fayettteville, AR 72701, USA; xqian@uark.edu

**Keywords:** atom transfer radical polymerization, flux, oscillating magnetic field, rejection, surface modification

## Abstract

Ultrafiltration membranes, that respond to an external magnetic field and local temperature have been developed. Surface-initiated activator-generated electron transfer (AGET) atom transfer radical polymerization (ATRP) has been used to graft poly(N-isopropylacrylamide) (PNIPAm) from the surface of 300 kDa regenerated cellulose membranes. The polymerization initiator was selectively attached to the entire membrane surface, only the outer membrane surface or only the inner pore surface. A superparamagnetic nanoparticle was attached to the end of the polymer chain. The DI water flux as well as the flux and rejection of bovine serum albumin were investigated in the absence and presence of a 20 and 1000 Hz oscillating magnetic field. In an oscillating magnetic field, the tethered superparamagnetic nanoparticles can cause movement of the PNIPAm chains or induce heating. A 20 Hz magnetic field maximizes movement of the chains. A 1000 Hz magnetic field leads to greater induced heating. PNIPAm displays a lower critical solution temperature at 32 °C. Heating leads to collapse of the PNIPAm chains above their Lower Critical Solution Temperature (LCST). This work highlights the versatility of selectively grafting polymer chains containing a superparamagnetic nanoparticle from specific membrane locations. Depending on the frequency of the oscillating external magnetic field, membrane properties may be tuned.

## 1. Introduction

Ultrafiltration is an established pressure-driven membrane filtration process. These membranes are capable of retaining species in the size range 2000 to 500,000 Da. Rejected species include proteins, enzymes, DNA, virus particles, polymers and colloidal particles [1]. Though ultrafiltration is considered a size-based separation process, numerous studies have indicated the importance of membrane–solute interactions as well as solution conditions [2]. In fact, by tailoring the membrane pore surface properties, one can tune membrane selectivity [3,4].

More recently, there has been growing interest in developing membranes whose performance can be modulated by environmental conditions. These stimuli-responsive membranes respond to an external stimulus [5,6,7]. One could include the responsive groups in the bulk membrane polymer or incorporate the responsive groups after membrane formation [8]. It is this latter approach that is used here. Numerous external stimuli have been investigated, such as pH, ionic strength, temperature, light and electric fields. Here, our focus is on grafting magnetically responsive brushes from the surface of a base ultrafiltration membrane. Magnetically responsive polymer brushes can induce both localized heating and movement of the grafted polymer chains.

A few investigators have considered the development of magnetically responsive ultrafiltration membranes by modifying the base membrane after formation. Superparamagnetic and paramagnetic nanoparticles are attached to the surface of ultrafiltration membranes. In an oscillating magnetic field, these particles can induce movement due to magnetophoretic actuation and alignment [9,10,11,12,13]. In addition, in an oscillating magnetic field, the magnetic nanoparticle can induce heating. Several investigators have included a grafted nanostructure consisting of a thermo-responsive polymer which exhibits a lower critical solution temperature (LCST), such as poly(N-isopropylacrylamide) (PNIPAm). Heating by the nanoparticles can lead to the local temperature exceeding the LCST, which is 32 °C in deionized (DI) water. This causes the grafted nanostructure to dehydrate and collapse, which will lead to changes in the membrane barrier properties [14,15].

In recent years, significant efforts have been directed towards grafting PNIPAm to the surface of a base membrane structure. Many investigators have focused on developing ‘smart’ membranes whose properties can be modified by changing the conformation of the grafted nanostructure. As the temperature is increased above the LCST of the grafted nanostructure, the nanostructure dehydrates and collapses, while below its LCST, it is hydrated and swells [16,17,18,19,20,21,22]. Others have considered the antifouling properties of PNIPAm grafted membrane surfaces. These investigators have considered the ability to detach adsorbed foulants by switching the feed temperature above and below the LCST of the grafted polymer nanostructure [22,23,24,25,26,27,28,29].

Our approach to develop magnetically responsive ultrafiltration membranes is different. We have tethered a superparamagnetic nanoparticle to the end of the polymer chains that are grafted from the surface of the base membrane. In our earlier work, we used atom transfer radical polymerization (ATRP) to graft polymer chains from the surface of nanofiltration and microfiltration membranes, followed by attachment of a superparamagnetic nanoparticle to the chain end. In a magnetic field, the particles experience a force and torque. Further, in an oscillating magnetic field, the particles can induce heating. By using PNIPAm as the polymer chain, heating induced in an oscillating magnetic field can cause the grafted PNIPAm nanostructure to collapse at temperatures above its LCST [30,31,32,33,34,35,36].

In our earlier work, we highlighted the importance of controlling the location of initiator immobilization prior to ATRP [37]. Often, if the initiator attaches to the pore surface, subsequent polymerization can lead to pore plugging. In this work, regenerated cellulose ultrafiltration membranes with a nominal molecular weight cut-off (NMWCO) of 300 kDa have been modified using ATRP. Three different strategies for initiator immobilization have been considered. In the first, the initiator is immobilized throughout the membrane inner pore surface and outer surface. The second strategy involves blocking the membrane pore surface by filling the pores with glycerol during initiator immobilization. In this way, grafting from the pore surface is suppressed. Finally, we attempt to immobilize the initiator only to the inner pore surface and not the outer membrane surface. After polymerization, a superparamagnetic nanoparticle is attached to the chain end and membrane performance is evaluated in an oscillating magnetic field. The frequency of the oscillating magnetic field is chosen to either maximize movement of the PNIPAm chains or induce heating of the PNIPAm chains, and hence collapse of the grafted nanostructure.

## 2. Materials and Methods

### 2.1. Materials

All reagents used were American Chemical Society (ACS) reagent grade or higher unless otherwise specified. Methanol, glycerol and acetonitrile were purchased from MilliporeSigma (Billerica, MA, USA). Triethylamine (TEA), 4-dimethylaminopyridine (DMAP), N,N,N′,N″,N″-pentamethyl diethylenetriamine (PMDETA, 99%), sodium phosphate monobasic (99%), sodium phosphate dibasic (99%), copper (I) bromide and copper (II) bromide (CuBr and CuBr_2_, 99.999% trace metal basis) were purchased from Sigma-Aldrich (Munich, Germany). N-isopropylacrylamide (NIPAm, >98%, stabilized with mequinol) was procured from TCI America (Boston, MA, USA). α-bromoisobutyryl bromide (BIB, 98%) was purchased from Alfa-Aesar (Ward Hill, MA, USA). 1,2-Epoxy-5-hexene was purchased from Acros Organic (98%, Pittsburgh, PA, USA). Bovine serum albumin (BSA, biotechnology grade) and L-(+)-ascorbic acid (AA) were purchased from Amresco (Solon, OH, USA). 2,2′-bipyridine (Bpy) was purchased from BeanTown Chemical (Hudson, NH, USA). Iron oxide superparamagnetic nanoparticles with a 15 nm core diameter and a 5 nm coating layer functionalized with amine groups were purchased from Ocean Nanotech (San Diego, CA, USA). The deionized (DI) water used in the present investigation was obtained from a Thermo Fisher 18 MΩ Barnstead Smart2Pure system (Schwerte, Germany). Commercially available regenerated cellulose membrane discs (44.5 mm diameter) with a nominal molecular weight cut-off of 300 kDa were obtained from MilliporeSigma.

### 2.2. Membrane Modification

**Scheme 1:**
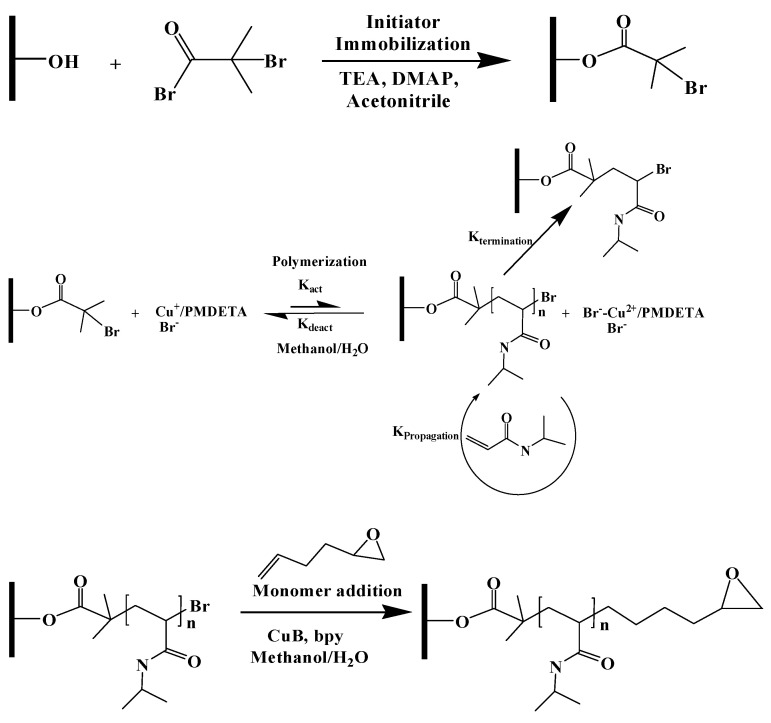

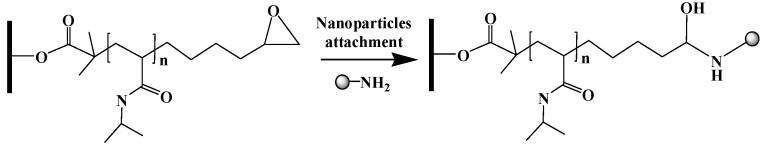
Membrane modification protocol.

Scheme 1 provides the membrane modification protocol. The first step was initiator immobilization. Three strategies were used: immobilization on the outer membrane surface and inner pore surface, immobilization only on the outer membrane surface and immobilization on the inner pore surface only. The second step was atom transfer radical polymerization, whereby PNIPAm chains were grown from the membrane surface. Next, monomer addition (atom transfer radical addition) was conducted to add 1-2 epoxy-5-hexane to the PNIPAm chain ends. Finally, the amine functionalized superparamagnetic nanoparticles were attached via epoxide ring opening.

#### 2.2.1. Initiator Immobilization

The membranes discs were washed three times in 25 mL of methanol for 20 min and then washed for 30 min in DI water. Acetonitrile was dried over activated molecular sieves prior to use. A washed membrane disc was rinsed 3 times with dry acetonitrile (20 mL) for 20 min. The reactive initiator immobilization solution was prepared by adding 100 mM BIB, 100 mM TEA and 5 mM DMAP to 20 mL of acetonitrile.

- Initiator immobilization on outer surface and inner pore surface

The membrane was transferred to the immobilization solution, then quickly sealed in the reaction vessel and the reaction was allowed to proceed at room temperature.

- Initiator immobilization on outer membrane surface only

The membrane disc was placed in a stirred cell (8050, MilliporeSigma) and the feed reservoir filled with glycerol. The feed pressure was increased to 1.5 bar and filtration continued until 5 mL of permeate was obtained. The membrane was then removed from the stirred cell and incubated in glycerol overnight. Next, the membrane surface was patted dry with a Kimwipe and then with a roller wiper in order to remove glycerol from the surface. The membrane was then placed on a glass plate, and an ethylenepropylenediene monomer (EPDM) gasket with a circular cutout 35 mm in diameter was centered on the sample. A piece of high-density polyethylene (HDPE) with dimensions matching the gasket was placed on top of the setup and the assembly was secured with binder clips. The initiator immobilization solution was added on top of the membrane and the reaction was allowed to proceed at room temperature.

- Initiator immobilization on internal pore surface of membrane

The outer surface of the barrier layer of the membrane was coated with glycerol. The membrane was then placed in a stirred cell (8050) with the functional layer facedown, as shown in Figure S1. The reactive monomer solution was added to the stirred cell and the reaction was allowed to proceed at room temperature.

For all three strategies, a reaction time of 1 min was investigated based on our earlier work [37]. Next, the reaction was quenched with water. The membrane was washed in a 1:1 (*v/v*) methanol/water mixture 3 times for 1 h. The modified region was cut out with a 25 mm punch and then incubated overnight in water.

#### 2.2.2. Activator Generated by Electron Transfer (AGET) ATRP

Surface-initiated activator-generated electron transfer (AGET) ATRP was used to graft N-isopropylacrylamide (NIPAm), from the initiator sites on the membrane surface [37]. The reaction solution was prepared by adding (NIPAm) (0.08 M), CuBr_2_ (0.8 mM) and PMDETA (2.4 mM) to a 1:9 (*v/v*) methanol/water mixture. Next, ascorbic acid at a concentration of 0.8 mM was added. The color of solution changed from blue to light purple. The reaction solution was introduced to the reaction vessel and the initiator functionalized membrane was added to the solution. The reaction vessel was placed on a shaker table and the reaction was allowed to proceed for 1, 2, 3 or 4 h for membranes with initiator immobilized on the inner pore and outer membrane surface, as well as the outer membrane surface only. For initiator immobilization on the inner pore surface only, 1- and 2-h polymerization times were investigated. After the specified reaction time had elapsed, the polymerization was terminated by adding the membrane into a quenching solution consisting of CuBr_2_ (0.22 M) and PMDETA (0.06 M) in a 1:1 (*v/v*) methanol/water mixture. The membranes were removed and transferred to the wash solution consisting of a 1:1 (*v/v*) methanol/water mixture for 2 h. The membranes were again washed and then stored in DI water for 1 h.

#### 2.2.3. Monomer Addition

The PNIPAm grafted membrane was placed in a Schlenk flask equipped with rubber stoppers. The flask was sealed with parafilm, evacuated and backfilled with argon three times. 1-2 epoxy-5-hexane (17.7 mM) and bpy (28.8 mM) were dissolved in a 1:1 (*v/v*) methanol/water mixture and purged with nitrogen for 30 min. Next, copper (I) bromide (5.58 mM) was added to the solution with rapid stirring under argon for 15 min. Finally, 30 mL of the reaction solution was added into the Schlenck flask that contained the modified membrane and kept at 50 °C for 24 h. After the reaction, the membrane was washed with a 1:1 (*v/v*) mixture of methanol/water 3 times for 1 h. The membrane was then immersed in water overnight.

#### 2.2.4. Nanoparticle Attachment

The membrane was rinsed and incubated in 20 mM phosphate buffer at pH 12 for 30 min. The reaction solution was prepared using 15 μL of the nanoparticle feedstock in 20 mL of 20 mM phosphate buffer at pH 12. The membrane was incubated in the reaction solution for 48 h on a shaker under gentle shaking at room temperature. Finally, the membrane was rinsed 3 times with water. The membrane was stored in water prior to use.

### 2.3. Membrane Characterization

#### 2.3.1. Degree of Grafting

Base membrane coupons were rinsed and dried overnight in a vacuum oven at 40 °C. The dried weight of the membrane was recorded. After modification, the membrane coupon was washed in DI water and then dried overnight in a vacuum oven at 40 °C. Three membrane coupons were modified at each condition. The membrane coupon was then weighed again. The degree of grafting was calculated from the following expression:

(1) $$D.G.=\frac{w_{1}-{\;w}_{0}}{A_{m}}$$

where, w_0_ and w_1_ are the masses of the unmodified and modified membrane respectively, while A_m_ stands for the area of the membrane used in the present investigation. Each value of the degree of grafting represents the average of three readings obtained for the three membranes modified at each condition. In all cases, the three readings were within 10% of each other. Since regenerated cellulose membranes are hygroscopic, it is critical to standardize the time between drying and weighing the membrane. Consequently, all membranes were weighed 30 min after being taken out of the oven.

#### 2.3.2. Contact Angle Measurement

The membrane coupon was dried in a vacuum oven at 40 °C overnight. Contact angle measurements were conducted at room temperature with DI water using a Future Digital Scientific, model OCA15EC (Garden City, NY, USA) contact angle goniometer. The droplet size was 2.0 µL. The droplet was dispensed at a speed of 0.5 µL/s. The contact angle was determined using the circle fitting method. Each value is the average of 5 repeat measurements.

#### 2.3.3. Attenuated Total Reflectance Fourier-Transform Infrared Spectroscopy (ATR-FTIR)

ATR-FTIR spectroscopy provides qualitative information about functional groups at the top, approximately 2 µm of the membrane. Data were collected using an IR Affinity instrument (Shimadzu, Columbia, MD, USA) with a horizontal ZnSe accessary. ATR-FTIR spectra were averaged over 100 scans covering a range of 1150–3650 cm^−1^. Prior to analysis, the membranes were dried overnight in a vacuum oven at 40 °C.

#### 2.3.4. Atomic Force Microscopy (AFM)

The surface topography of the membrane at room temperature was measured using a Dimension Icon AFM (Bruker, Santa Barbara, CA, USA). The NanoScope V815R3sr1 program was used to run the AFM and the NanoScope Analysis program was used to analyze the results. The ScanAsyst mode was used to image the membranes in air using an etched silicon tip on a nitride lever, which was coated with a 100 nm aluminum layer. Membrane coupons were dried overnight at 40 °C prior to analysis. The nominal spring constant of the cantilever used was 0.4 N/m and 70 kHz, respectively. A standard scan rate of 1 Hz with 512 samples per line was used. The measured heights of the images were then flattened in order to obtain the final images.

### 2.4. Membrane Performance

All testing was conducted at room temperature in dead-end filtration mode using a stirred cell (8010, MilliporeSigma). Prior to flux measurement, the membranes were soaked in methanol for 15 min, then DI water for 15 min. Pressurized nitrogen was used to drive the feed through the membrane. The feed pressure was 10.3 kPa (1.5 psi). The DI water flux was determined after 15 min of operation. Our results indicated that by this time, the flux had reached its steady state value. Feed streams consisting of BSA (1 mg mL^−1^) were also tested. The BSA was suspended in 100 mM Tris-HCl, pH 7.5 (Millipore-Sigma). All tests were conducted without stirring in the presence and absence of an oscillating magnetic field. The feed pressure was 10.3 kPa. Flux and rejection data were collected after 15 min of operation. During this period, a pseudo steady-state flux was obtained, where the flux did not vary much with time. BSA rejection was calculated as follows:

(2) $$R=1-\frac{C_{p}}{C_{f}}\times 100\;\%$$

where R is the percentage of rejection and C_p_ and C_f_ correspond to the permeate and feed concentrations.

An oscillating magnetic field was generated using a custom-built system [30,31]. The stirred cell was placed between two solenoids. A computer-controlled programmable logic controller (PLC, Click Koya Automation Direct, Cumming, GA, USA) controlled the two solenoids by alternatively activating two solid-state relays. This enabled us to set the frequency of the alternating magnetic field. The solenoids were powered by an Agilent Technologies (Santa Clara, CA, USA) 20 V/25 A power supply. The solenoids were placed on opposite sides of the filtration cell so that the magnetic field was parallel to the barrier layer of the membrane.

The oscillation frequency of the magnetic field was set at 20 or 1000 Hz, where the current was set 2 A and 8 V. Our previous studies indicated that 20 Hz maximizes movement of the superparamagnetic nanoparticles used here [30,31]. As higher frequencies induce heating, 1000 Hz was also tested [36]. Insulated foam was placed between the solenoids and filtration cell in order to prevent heat transfer to the cell from the solenoid.

As we have shown in earlier studies [36], this is essential to ensure that an increase in the bulk water temperature is suppressed and will not lead to modified fluxes due to changes in viscosity. Further, the frequency we choose, 1000 Hz, is relatively low for applications focused on magnetically induced heating. This is important in order to ensure that heating effects are localized to the grafted polymer chains and do not lead to an increase in the bulk water temperature.

A Mettler Toledo PL 602~S (Columbus, OH, USA) balance connected to a computer was used to measure the weight of the permeate, which in turn was used to calculate the permeate flux. The experimental set-up is shown in Supplementary Figure S2.

## 3. Results

### 3.1. Membrane Characterization

#### 3.1.1. Degree of Grafting

**Figure 1:**
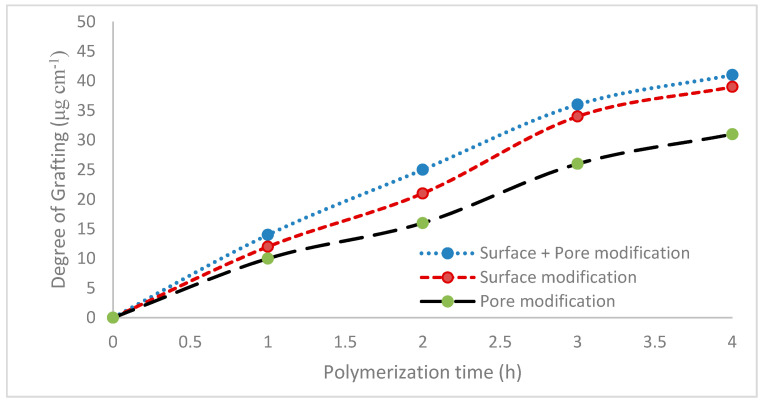
Variation of degree of grafting with polymerization time for the three initiator immobilization strategies. The dotted, small dashed and loner dashed lines represent pore modification, external surface modification and external surface and pore modification, respectively.

Figure 1 shows the degree of grafting for the three initiator immobilization strategies. As can be seen, the degree of grafting increases with polymerization time. This is expected as the amount of grafted material increases with polymerization time. Figure 1 also indicates that the increase in the degree of grafting is approximately linear, especially at lower polymerization times. Consequently, this is a controlled polymerization reaction.

The increase in degree of grafting for a given polymerization time depends on the initiator immobilization strategy. It is lowest for initiator immobilization on the membrane inner pore surface, even though the inner pore surface represents a significant fraction of the total membrane surface area. The degree of grafting is greatest for initiator immobilization on the inner pore and outer membrane surface. In the case of initiator immobilization on the inner pore surface, there is likely to be a significant resistance for NIPAm diffusion to the growing polymer chains during polymerization. This would explain the lower degree of grafting compared to the two other immobilization strategies. Initiator immobilization on the inner pore and outer membrane surface provides the maximum number of initiation sites for polymerization, explaining why the highest degree of grafting is obtained for this initiator immobilization strategy.

#### 3.1.2. Contact Angle

**Figure 2:**
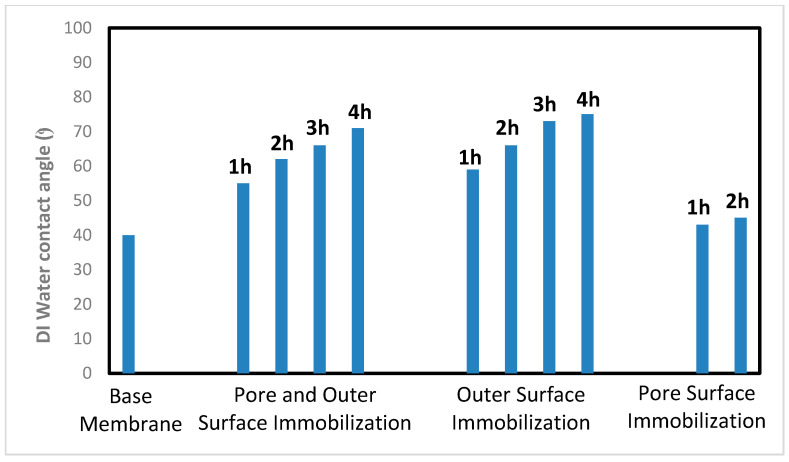
DI water contact angle for the base membrane and membranes modified using the three initiator immobilization strategies. Results are shown for the different polymerization times investigated and represent the average of 5 repeat readings with a range of ±10%.

Static contact angle results are shown in Figure 2. As can be seen, the base membrane is very hydrophilic. Surface modification leads to an increase in the contact angle. The contact angle increases with increasing polymerization time as the level of surface coverage by PNIPAm increases. This is expected as the cellulosic surfaces are far more hydrophilic than PNIPAm. The figure also indicates that when initiator immobilization is limited to the inner membrane pore surface, the contact angle is very similar to the base membrane. This is expected as the contact angle measurement is based on the outer membrane surface.

#### 3.1.3. ATR-FTIR

**Figure 3:**
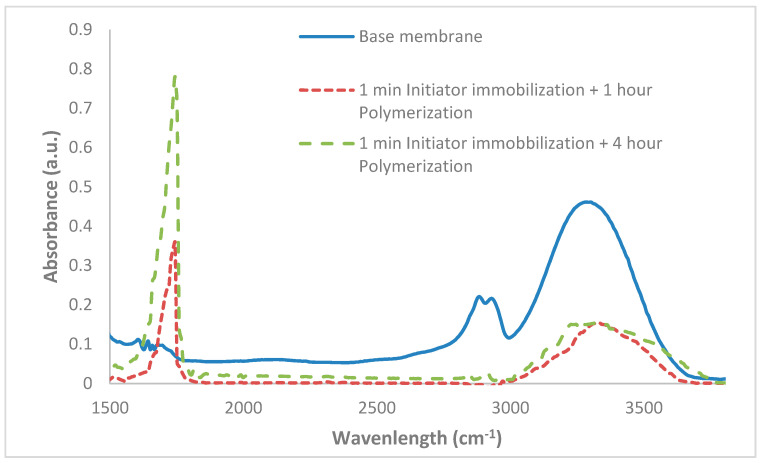
Fourier Transformed Infrared (FTIR) spectra for base and modified membranes. Initiator was immobilized on the inner pore and outer membrane surface, followed by polymerization for 1 and 4 h.

FTIR spectra were obtained for base and modified membranes. Initiator immobilization was conducted on the outer and inner pore surface. Spectra are shown in Figure 3 for the base membrane and for 1- and 4-h polymerization times. Comparing the spectra for the base and modified membranes indicates a decrease in the broad peak at ca 3278 cm^−1^ for the modified membranes. This peak may be attributed to the stretching frequency of –OH groups in the base membrane. Upon modification, the peak decreases as a PNIPAm nanostructure is now present on the outer membrane surface. However, a new peak is detected corresponding to the carbonyl groups present in the grafted PNIPAm nanostructure. The intensity of the peak increases for longer polymerization times due to the fact the that the thickness of the grafted PNIPAm layer increases with increasing polymerization time.

#### 3.1.4. AFM Imaging

**Figure 4:**
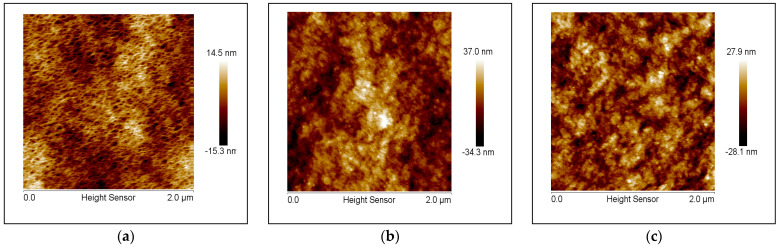
Atomic Force Microscopic (AFM) images of (**a**) base membrane, modified membranes after (**b**) 1-h polymerization and (**c**) 4-h polymerization. The initiator was immobilized on the inner pore and outer membrane surface.

AFM images of base and modified membranes are provided in Figure 4. Figure 4a shows the base membrane while Figure 4b,c show images for modified membranes. The modification conditions were analogous to Figure 3. The initiator was immobilized on the inner pore and outer membrane surface followed by polymerization for 1 (Figure 4b) and 4 (Figure 4c) hours. Figure 4b,c indicates the presence of nanoparticles. The average particle size was found to be ~25–30 nm, in keeping with the manufacturer’s specifications. Comparing Figure 4b,c, it appears that qualitatively, the number of attached nanoparticles is similar. The result highlights our ability to vary polymer chain length by increasing polymerization time while maintaining a similar chain density.

This study has focused on our ability to control the structure and location of the PNIPAm chains that are grafted from the membrane surface. Minimizing the polydispersity of the grafted chains is important in order to observe a sharp transition in the conformation of these chains due to changes in environmental conditions. Figure 1 indicates that we have a high degree of control over the polymerization reaction. At longer polymerization times, the increase in the degree of grafting deviates from linear growth. This is not unexpected as the likelihood of chain termination events will increase. In addition, the growing chain ends could be sterically hindered within the grafted nanostructure.

It is important to remember that in order to determine the degree of grafting, the membrane must be dried before any modification and after modification in order to obtain an accurate weight of the grafted nanostructure. Drying regenerated cellulose ultrafiltration membranes can lead to collapse of the pore structure. The AGRT ATRP modification protocol developed here avoids the need to dry the membrane. Thus, for membranes that were modified in order to determine the degree of grafting, the membrane pore structure is most likely less open than the modified membranes that were tested for filtration performance. Nevertheless, Figure 1 and Figure 2 together provide evidence of our ability to graft relatively uniform chains from the inside pore or outer membrane surface.

Figure 3 and Figure 4 indicate changes on the outer membrane surface. Neither FTIR spectra nor AFM images of the membrane surface provide evidence of modification of the inner pore surface. Consequently, results are only reported for initiator immobilized on the inner and outer membrane surface. FTIR spectra and AFM images for initiator immobilized on the outer membrane surface only are identical to Figure 3 and Figure 4, respectively. For initiator immobilized on the inner pore surface, FTIR spectra and AFM images appear similar to the base membrane.

### 3.2. Membrane Performance

#### 3.2.1. Water Flux

Table 1, Table 2 and Table 3 provide water flux data for initiator immobilized on the inner pore and outer membrane surface, outer membrane surface and inner pore surface. The base membrane water flux was 630 L m^−2^ h^−1^. For initiator immobilization on the inner pore and outer membrane surface (Table 1), data are presented for 1-h polymerization. As can be seen for 1-h polymerization, an order of magnitude decrease in flux was observed compared to the base membrane. For longer polymerization times, no flux was recorded for a feed pressure of 10.3 kPa. Similarly, data for initiator immobilized on the inner pore surface (Table 3) are presented for 1- and 2-h polymerization times only. Again, an order of magnitude decrease in flux is observed compared to the base membrane. For 3- and 4-h polymerization times, the flux at 10.3 kPa was not measurable.

The results provided in Table 1, Table 2 and Table 3 are sequential. First, the water flux was measured in the absence of an oscillating magnetic field. Next, the membrane was subjected to a 20 Hz oscillating magnetic field followed by a 1000 Hz field. Finally, the membrane was retested in the absence of an oscillating magnetic field. All the results indicate that the observed changes in water flux are reversible. When the magnetic field is removed, the initial water flux for the modified membrane is regained. Further, the longer the polymerization time, the lower the water flux. This is not unexpected as longer polymerization times lead to a greater thickness of the grafted nanostructure, which will lead to a larger resistance to permeate flow.

The results in Table 1 indicate that a 20 Hz oscillating magnetic field has no effect on the water flux. However, an increase in water flux is observed for a 1000 Hz oscillating magnetic field. The results in Table 2 suggest that for initiator immobilization on the outside membrane surface, a 20 and 1000 Hz field have little effect on the water flux. However, Table 3 indicates that for initiator immobilization on the inner pore surface, a 20 Hz field has little effect on the water flux, but a 1000 Hz field leads to an increase in water flux.

#### 3.2.2. BSA Filtration

BSA filtration was conducted for initiator immobilized on the outer membrane surface and inner pore surface. The base membrane BSA flux was 580 L m^−2^ h^−1^. As expected, it is lower than the water flux. In agreement with the water flux data (Table 1), reproducible data could not be obtained for initiator immobilized on the inner pore and outer membrane surface. Table 4 provides BSA flux data for initiator immobilized on the outer membrane surface, while Table 5 provides data for initiator immobilized on the inner pore surface. As was observed for the water flux, the observed changes in flux are reversible. Further the BSA flux decreases with increasing polymerization times. When the magnetic field is removed, the initial BSA flux for the modified membrane is recovered. Further, Table 4 indicates that for initiator immobilized on the outer membrane surface, the presence of a 20 and 1000 Hz oscillating magnetic field has little effect on the BSA flux. Similarly, analogous to the water flux data shown in Table 3, when initiator is immobilized on the inner pore surface, a 20 Hz field leads to little change in the BSA flux, but a 1000 Hz field leads to an increase in flux.

**Figure 5:**
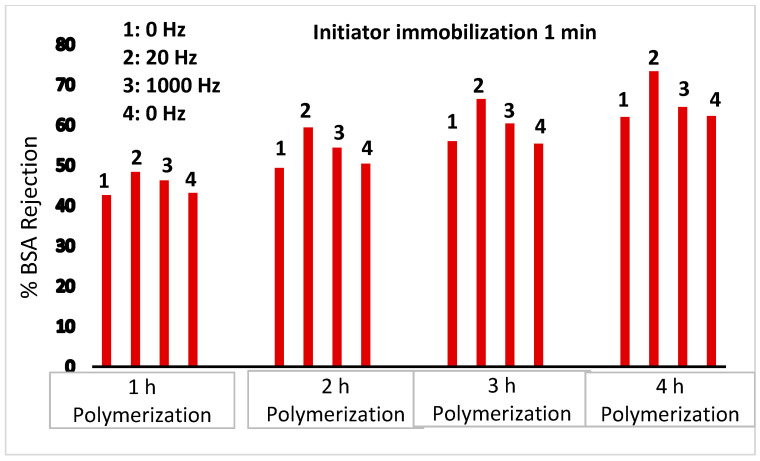
Variation of BSA rejection for outer membrane surface modification. Results are shown in the absence and presence of a 20 and 1000 Hz oscillating magnetic field.

**Figure 6:**
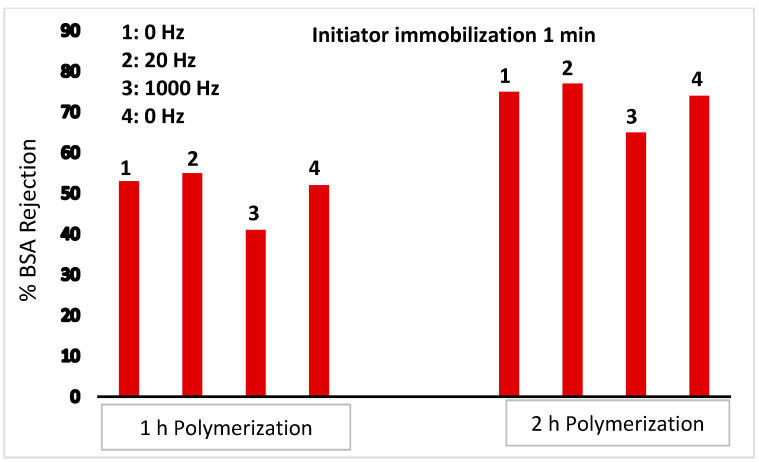
Variation of BSA rejection for inner membrane pore surface modification. Results are provided in the absence and presence of a 20 and 1000 Hz oscillating magnetic field.

BSA rejection data are provided in Figure 5; Figure 6 for initiator immobilized on the outer membrane surface and inner pore surface, respectively. Both figures indicate that as the polymerization time increases, so does the observed BSA rejection. Figure 5 indicates that a 20 Hz field leads to the greatest increase in rejection for all polymerization times. A 1000 Hz field leads to a lower increase in rejection compared to no magnetic field. However, Figure 6 indicates a different result. A 20 Hz field has little effect on BSA rejection, but a 1000 Hz field leads to a decrease in the observed BSA rejection. In all cases, the result is reversible. When the magnetic field is removed, the initial BSA rejection for the modified membrane is regained.

## 4. Discussion

In this work, we have, for the first time, attached a superparamagnetic nanoparticle to the end of a PNIPAm chain that is grown from the ultrafiltration membrane surface. By controlling the location of grafting, we can probe the effect of changes in the conformation of the PNIPAm chains due to an external stimulus for polymer chains grafted from different membrane surfaces. Here, we have investigated the effect of an external oscillating magnetic field.

Superparamagnetic nanoparticles are unique in that they are single domain nanoparticles. Consequently, they behave like a paramagnet with a giant magnetic moment in the presence of a magnetic field [38]. For superparamagnetic nanoparticles, the exchange coupling of the atomic magnetic moments is strong, resulting in alignment of the moments with each other. Further, creation of domain walls is energetically unfavorable, resulting in a single magnetic domain. At room temperature, in the absence of a magnetic field, the magnetic anisotropy energy is less than the thermal energy, leading to a random orientation of the giant moment in space. However, the giant moments will readily align in the direction of an external magnetic field. When an external field is removed, superparamagnetic nanoparticles display no remanence. Brownian and Néel relaxation result in the dispersion of the magnetic dipoles [39,40].

Brownian relaxation (μ_B_) describes the physical rotation of the particles as the magnetic moment within each particle randomizes with respect to neighboring particles. Néel relaxation (μ_N_) on the other hand describes the rotation of the magnetic moment within each particle due to this randomization process. Importantly, energy dissipation due to relaxation will lead to heating. Since the particles are tethered to PNIPAm chains, the heat induced can lead to an increase in temperature of the PNIPAM chains above their LCST, which will lead to collapse of the grafted nanostructure. The two relaxation times are determined from the following expressions:

(3) $$\tau_{B}=\frac{3V_{H}\eta }{k_{B}T}$$

(4) $$\tau_{N}=\tau_{0}\frac{3V_{H}\eta }{k_{\mathrm{BT}}}$$

where V_H_ and V_M_ are the hydrodynamic and magnetic volumes of the nanoparticles, η is the viscosity, K is the effective anisotropy constant, k_B_ is the Boltzmann constant, T is the absolute temperature and $\tau_{0}$ is constant. As can be seen, the two relaxation times will be different. For the nanoparticles used here (15 nm magnetic core, 5 nm coating), the calculated Néel relaxation time is much larger than 1 s and the Brownian relaxation time is in the order of 10^−3^ s.

In an external magnetic field, the superparamagnetic nanoparticle will experience a torque and a force, as given by Equations (5) and (6):

(5) $$\tau =\vec{\mu }⊗\;\vec{B}$$

(6) $$F=\left(\vec{\mu }\cdot \nabla \right)\;\vec{B}$$

where τ and F are the torque and force experienced by the nanoparticles and $\vec{\mu }$ and $\vec{B}$ are the magnetic moment of the nanoparticles and the external magnetic field. The torque results in rotation of the particles through Brownian and Néel relaxation, as described above. The force on the particles will lead to translational motion, and hence, mixing. As shown in our earlier work [30], translational motion is maximized at frequencies around 20 Hz. Higher frequencies will lead to greater heat generation. Thus, we have investigated the effect of a 20 and 1000 Hz oscillating magnetic field on membrane performance.

For initiator immobilized on the inner pore and outer membrane surface, Table 1 indicates that a large decrease in flux is observed, indicating significant pore blocking by the grafted nanostructure. When movement of the grafted polymer chains is maximized at 20 Hz, little change in permeate flux is observed. However, when induced heating is maximized at 1000 Hz, an increase in flux is observed. Here, we insulated the stirred cell to minimize heat transfer from the solenoids. Further, our previous work indicated that insulation in the stirred cell results in little increase in the bulk water temperature [36]. An increase in the water temperature will lead to a decreased viscosity and can also explain the increased permeate flux.

Collapse of the grafted PNIPAm chains in the membrane pores at temperatures above their LCST will also lead to an increase in membrane pore size and observed flux. This assertion is supported by our earlier studies that indicate an increase in the minimum size of particles found in the retentate of track-etched microfiltration membranes also grafted with PNIPAm chains [36]. In addition, the data in Table 3; Table 5 for initiator immobilized only on the membrane pore surface are consistent with this assertion. BSA rejection data in Table 5 indicate a decrease in BSA rejection at 1000 Hz for initiator immobilized on the inner pore surface, consistent with our earlier microfiltration studies [36].

Table 2 indicates that as the thickness of the PNIPAm nanostructure grafted from the outer surface of the membrane increases, the water flux decreases. It is likely that polymer chains grafted at the pore mouth will bridge across the pore entrance, leading to a narrowing of the pore mouth and hence a decrease in permeate flux. The effect will increase as the chain length increases with longer grafting times. The results for BSA filtration shown in Table 5 are in agreement with the water flux data.

BSA rejection data shown in Figure 5, however, suggest that for initiator immobilized on the outer membrane surface, the presence of a 20 Hz oscillating magnetic field leads to an increase in the observed rejection. However, no change in the rejection is observed for a 1000 Hz oscillating magnetic field. In a 20 Hz oscillating magnetic field, movement of the grafted PNIPAm chains will be the dominant effect. In our earlier work, we have shown that his movement can lead to break up of the concentration polarization boundary layer [30,31]. For nanofiltration membranes, this led to an increase in the observed rejection of partially rejected salts. The result observed here for ultrafiltration membranes is analogous. On the other hand, collapse of the grafted PNIPAm chains due to induced heating is expected to have little effect on BSA rejection for PNIPAm chains grafted from the outer membrane surface.

Taken together, the data indicate for that for ultrafiltration membranes, selectively grafting magnetically and thermally responsive polymer chains on the outer or inner pore surface of the membrane can lead to remote modulation of membrane performance. However, grafting PNIPAm polymer chains from the entire membrane surface limits the degree to which one can tune membrane performance.

In our earlier work, we grafted the same PNIPAm chains from the surface of nanofiltration and track-etched microfiltration membranes [36]. No attempt was made for surface-specific initiator immobilization. In the case of nanofiltration membranes, the very small pore size present results in little grafting on the pore surface. Diffusional and steric hindrance effects limit monomer and nanoparticle transport into the pores. For microfiltration membranes, given their large pore size (0.4 μm), PNIPAm grafted from the outer membrane surface had little effect on performance. Ultrafiltration membranes lie in between microfiltration and nanofiltration membranes. In this case, careful surface-selective grafting can lead to a high degree of performance modulation.

## 5. Conclusions

Design of responsive membranes offers the promise of tuning membrane performance based on external conditions. Here, we showed that incorporation of a superparamagnetic nanoparticle at the end of a thermo-responsive polymer chain can enable remote performance modulation using an external magnetic field. Movement of the polymer chains as well as collapse of the polymer chains at temperatures above their LCST can lead to different polymer conformations. These different conformations may be exploited to tune membrane performance.

Previous investigations have indicated the importance of controlling the three-dimensional structure of the grafted polymer chains. In particular, polymer chain density and length will affect the response of the grafted polymer chains to changes in the external magnetic field. Here, we highlighted the importance of controlling the location of surface-initiated polymerization. Grafting polymer chains selectively only from the outer surface of membranes or the inner pore walls can lead to much finer modulation of membrane performance compared to non-surface-selective modification.

## Figures and Tables

**Table 1:** Variation of water flux for inner pore and outer membrane surface modification. No flux was measured at 10.3 kPa for polymerization times greater than 1 h. Results are shown in the absence and presence of a 20 and 1000 Hz oscillating magnetic field. Average results are provided with a range of ±8 L m^−2^ h^−1^.

Flux (L m^−2^ h^−1^)				
Base Membrane	No Field	20 Hz	1000 Hz	No Field
630	58	58	73	58

**Table 2:** Variation of water flux for outer surface modification. Results are shown in the absence and presence of a 20 and 1000 Hz oscillating magnetic field. Average results are provided with a range of ±8 L m^−2^ h^−1^. The feed pressure was 10.3 kPa.

Polymerization Time (h)	Outer Surface Modification, Flux (L m^−2^ h^−1^)			
	No Field	20 Hz	1000 Hz	No Field
1	551	551	546	547
2	481	476	474	483
3	437	437	434	435
4	403	401	405	400

**Table 3:** Variation of water flux for inner pore surface modification. Results are shown in the absence and presence of a 20 and 1000 Hz oscillating magnetic field. Average results are provided with a range of ±8 L m^−2^ h^−1^. The feed pressure was 10.3 kPa.

Polymerization Time (h)	Inner Pore Surface Modification, Flux (L m^−2^ h^−1^)			
	No Field	20 Hz	1000 Hz	No Field
1	73	68	89	74
2	60	56	88	61

**Table 4:** Variation of bovine serum albumin (BSA) flux for outer membrane surface modification. Results are shown in the absence and presence of a 20 and 1000 Hz oscillating magnetic field. Average results are provided with a range of ±8 L m^−2^ h^−1^. The feed pressure was 10.3 kPa.

Polymerization Time (h)	Outer Surface Modification, Flux (L m^−2^ h^−1^)			
	No Field	20 Hz	1000 Hz	No Field
1	559	550	544	545
2	482	475	472	481
3	433	437	433	434
4	407	401	403	406

**Table 5:** Variation of BSA flux for inner membrane pore surface modification. Results are shown in the absence and presence of a 20 and 1000 Hz oscillating magnetic field. Average results are provided with a range of ±8 L m^−2^ h^−1^. The feed pressure was 10.3 kPa.

Polymerization Time (h)	Inner Pore Surface Modification, Flux (L m^−2^ h^−1^)			
	No field	20 Hz	1000 Hz	No Field
1	74	68	89	74
2	49	43	87	60

## Data Availability

The data presented in this study are available on request from the corresponding author.

## Supplementary Materials

The following are available online at https://www.mdpi.com/article/10.3390/membranes11050340/s1, Figure S1: Schematic diagram for initiator immobilization on internal pore surface of membrane title, Figure S2: Experimental set up for membrane performance evaluation.
