# Supplementary material for: Remote Performance Modulation of Ultrafiltration Membranes by Magnetically and Thermally Responsive Polymer Chains
Source: Membranes (Basel). 2021 May 4;11(5):340. doi: 10.3390/membranes11050340 (PMC8147820; doi:10.3390/membranes11050340)
Supplement: Supplementary file 1 [file membranes-11-00340-s001.zip › membranes-1176799-supplementary.pdf]

membranes

Supplementary Information: Remote Performance Modulation of Ultrafiltration Membranes by Magnetically and Thermally Responsive Polymer Chains

Arijit Sengupta, Anh Vu, Xianghong Qian and S. Ranil Wickramasinghe

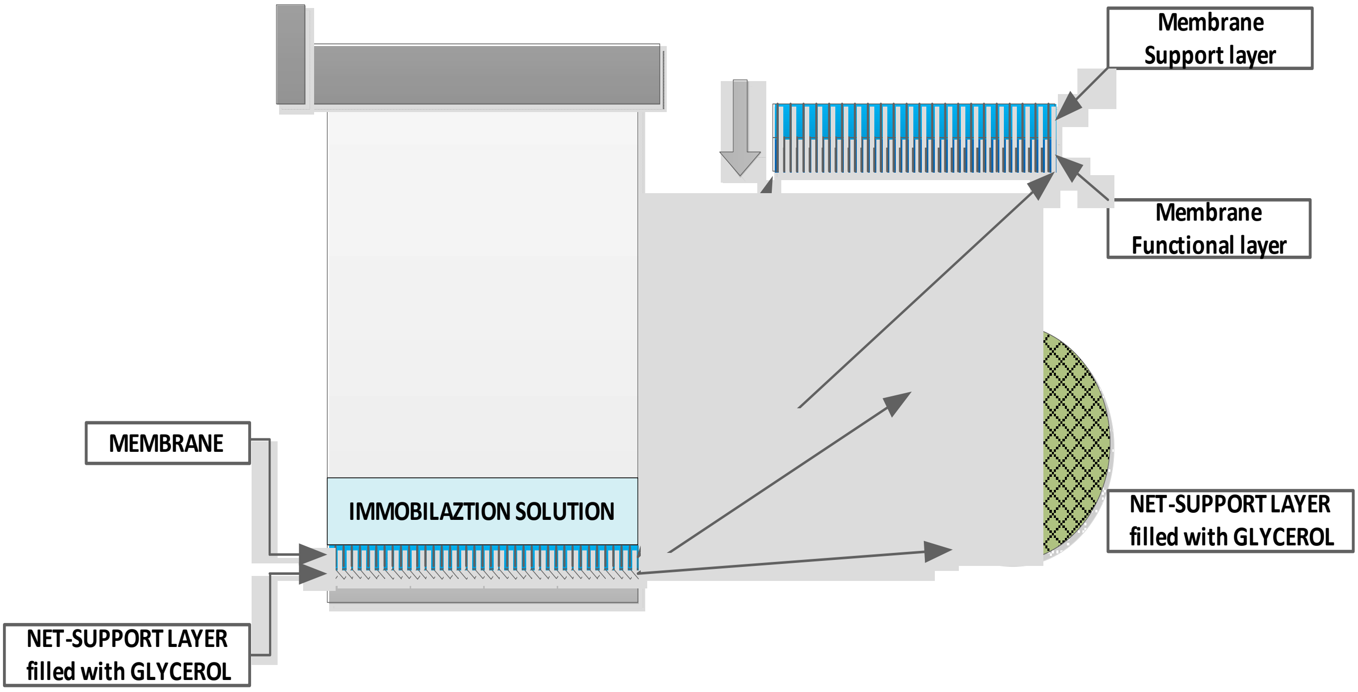

Figure S1. Schematic diagram for initiator immobilization on internal pore surface of membrane.

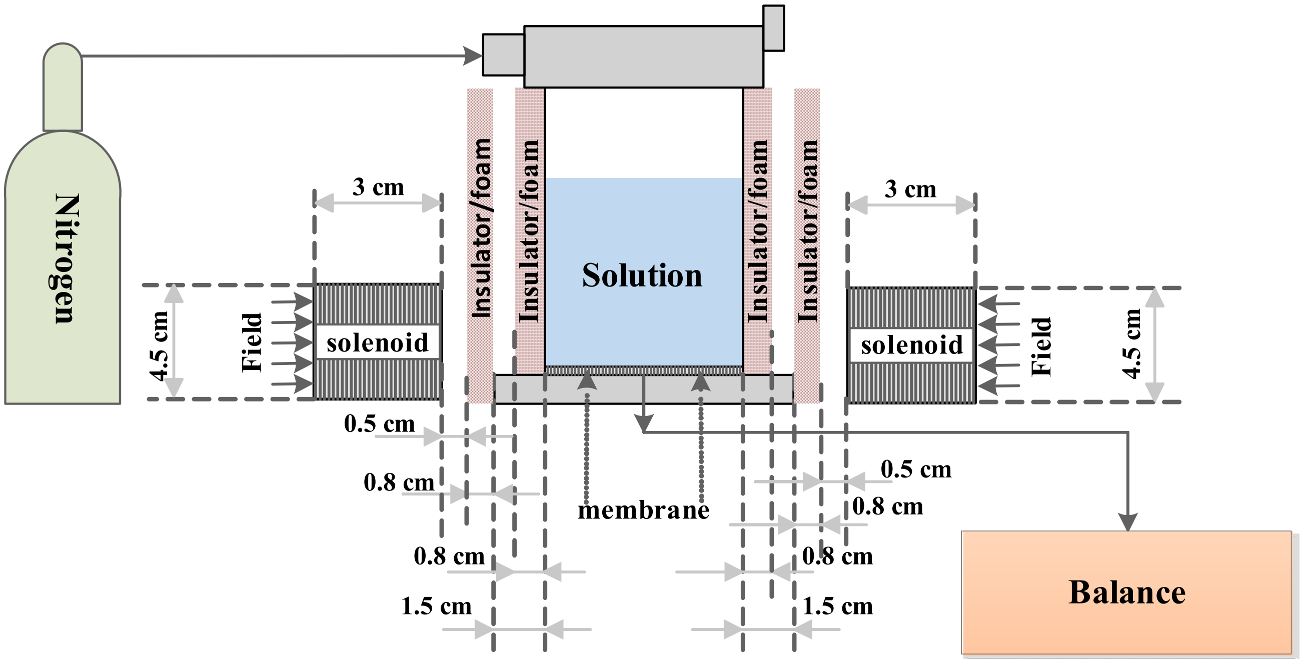

Figure S2. Experimental set up for membrane performance evaluation.
